# Six HIT Sessions Improve Cardiorespiratory Fitness and Metabolic Flexibility in Insulin Resistant and Insulin Sensitive Adolescents with Obesity

**DOI:** 10.3390/ijerph191710568

**Published:** 2022-08-25

**Authors:** Juliana Monique Lino Aparecido, Marcelo Luis Marquezi, Hellyelson Lopes de Omena Couto, Thais Miriã da Silva Santos, Alison Fabiano Cunha Cruz, Nayara Barbosa Lopes, Marcelo Santin Cascapera, Vivian Bertoni Xavier, Cristiane Kochi, Vera Lúcia dos Santos Alves, Antonio Herbert Lancha

**Affiliations:** 1 Laboratory of Physical Education Research (LAPEF), University City of Sao Paulo (UNICID), Sao Paulo 05508-030, Brazil; 2 Pediatric Cardiology Group, Department of Pediatrics, Childcare of Irmandade da Santa Casa de Misericórdia de São Paulo (ISCMSP), Sao Paulo 01221-010, Brazil; 3 Santa Casa de Sao Paulo School of Medical Sciences, Sao Paulo 01221-010, Brazil; 4 Molecular Medicine Laboratory, Department of Pediatrics, Childcare of Irmandade da Santa Casa de Misericórdia de São Paulo (ISCMSP), Santa Casa de Sao Paulo School of Medical Sciences, Sao Paulo 01221-010, Brazil; 5 Physiotherapy Service, Irmandade da Santa Casa de Misericórdia de São Paulo, Postgraduate—Universidade de Mogi das Cruzes (UMC), Sao Paulo 01221-010, Brazil; 6 Laboratory of Clinical Investigation Experimental Surgery (LIM 26), Clinic’s Hospital of Medical School, University of Sao Paulo, Sao Paulo 05508-030, Brazil

**Keywords:** obesity, insulin-resistant, adolescents, high-intensity interval training

## Abstract

To evaluate the effect of high-intensity interval training (HIT) on the cardiorespiratory performance and substrate oxidation pattern in insulin-resistant and insulin-sensitive obese adolescents. Methods: We recruited 25 obese adolescents in three schools, and trained them in six HIT sessions, comprising of six series at 100% and recovery at 50% peak velocity (Vpeak). For the evaluation, the participants were divided into two groups: insulin-resistant (IR, n = 12; HOMA index ≥3.16) and insulin-sensitive (IS, n = 13). All participants underwent cardiopulmonary and indirect calorimetry testing. We compared the effects of HIT before and after the intervention among the two groups. The data were analyzed using Student’s *t* and Mann–Whitney (intergroup comparisons) and Student’s *t* and Wilcoxon (pre- and post-training comparisons) tests; and Cohen’s *d* (influence of HIT). Results: There was a significant post-training increase in Vpeak, oxygen consumption (VO2), velocity (V), and heart rate (HR) at the exertion intensity at the first ventilatory anaerobic threshold (VAT1) in both groups (*p* < 0.05; *d* < 0.02). The exercise promoted changes in substrate oxidation rates of the groups, with an increase in carbohydrate oxidation (CHOox) for both IR (*p* = 0.064) and IS (*p* = 0.034). Conclusion: Six HIT sessions improved cardiorespiratory performance in both groups and increased CHOox in insulin-sensitive obese adolescents, suggesting its utility for increasing physical fitness and controlling glycemia in these population groups.

## 1. Introduction

Considered an inflammatory disease, obesity has been a worldwide epidemic, regardless of sex, age, and socioeconomic status [1,2,3]. Its inflammatory mechanisms are related to the expression of adipokines (tumor necrosis factor-alpha, interleukin 6, interleukin 1 beta, leptin, resistin, and plasminogen activator inhibitor) [4,5]. This low-grade chronic inflammation is directly related to the development of type II diabetes, atherosclerosis, and metabolic syndrome [2,3]. In addition, the increase in visceral fat contributes to the reduction of anti-inflammatory adipokines such as adiponectin and impaired glucose translocation to the plasma membranes [6].

Overweight young people are more susceptible to having metabolic disorders, since the accumulation of body fat, especially in the abdominal region, generates an increase in circulating fatty acids in the bloodstream, which consequently impairs insulin signaling and leads to a reduction in blood flow, sensitivity of receptors and tissue response to cellular actions that are mediated by this hormone [3,6]. Elevated uric acid levels (hyperuricemia) are also implicated in the pathogenesis of obesity and have been linked to impaired glucose tolerance, impaired fasting glucose, and type 2 diabetes [3].

There is a number of methods for the prevention and treatment of insulin resistance in obese individuals. Physical training is one of the most widely used non-pharmaceutical approaches [7,8,9,10,11]. Studies in adults have shown improved insulin sensitivity with regular physical activity, although the mechanisms involved in these adaptations in children and adolescents have not yet been fully elucidated [7,12].

It is recommended that children perform 30–60 min of moderate-to-high intensity exercise, 3–4 times per week [9]. In general, these programs take at least 12 weeks (36–48 sessions). However, lack of time, difficulty accessing specific facilities, and low motivation are some of the barriers to low adherence, i.e., failure to engage in regular exercise [10]. In this context, high-intensity interval training (HIT) is available, characterized by short bouts of vigorous activity interspersed with rest or low-intensity exercise [11], with the advantage of improving cardiorespiratory fitness and energy substrate oxidation in adults in six sessions [12,13]. Moreover, HIT when compared to traditional endurance training or moderate-intensity continuous exercise (MICT), invariably related to ventilatory anaerobic thresholds (VATs), can produce similar and even superior changes in physiological and physical performance and health-related outcomes, but with a sustainably reduced duration and volume of exercise [14].

HIT is an effective method for improving cardiorespiratory fitness in adolescents, regardless of body composition. Notably, meta-regression analysis identified that extended high-volume HIT programs are equally effective for short-term low-volume HIT programs [15]. Studies in adults suggest that HIT increases insulin sensitivity, a finding that has prompted further investigations in obese individuals [7,16]. However, there are no studies assessing the effect of HIT on cardiorespiratory fitness and energy substrate oxidation in insulin-resistant and insulin-sensitive obese adolescents [8].

We hypothesize that HIT might improve carbohydrate and fat metabolism, and also that the energy substrate oxidation pattern might be different in individuals who are insulin-resistant and insulin-sensitive, with those with higher resistance benefitting more from exercising. This rationale inspired us to perform this study, in which the objective was to evaluate the effects of HIT on the cardiorespiratory performance and substrate oxidation of insulin-resistant and insulin-sensitive obese adolescents.

## 2. Materials and Methods

### 2.1. Trial Design, Setting and Ethics

This is a pre-post clinical study on the effects of the same intervention (exercising) in two different groups of patients (insulin-resistant and insulin-sensitive obese adolescents), comparing the variables before the training with the same cardiorespiratory variables after exercising. The study could not be randomized or have a placebo or control group, since exercising was offered, as a recommended non-pharmacological treatment for obesity, for all obese adolescents in all institutions. Not offering the intervention was not considered ethically acceptable. The physical therapists performing the evaluations were blinded for the insulin resistance condition of each patient (allocation).

The study was evaluated and approved by the local research ethics committee (CAEE 34634414.5.0000.5479) of the Pediatric Endocrinology Clinic of the university hospital where it was performed. After ethical approval, it was registered in the ClinicalTrials.gov database (NCT03042234). All participants’ legal guardians signed a free and informed agreement/consent form for the inclusion in the study, the anthropometric measures, and the intervention (exercise training). We reported the study methods and results in accordance with the CONSORT reporting guideline [17].

### 2.2. Sample Size

The sample size was calculated aiming to achieve a 23% difference in the carbohydrate outcome (13 participants per group), and considering a 5% chance of type 1 error and 80% power, according to the study of Barker et al. [18].

### 2.3. Participants

We recruited adolescents for this study in a hospital and three municipal schools located in the metropolitan region of a Brazilian capital city, from May 2017 to January 2018. We worked with school and hospital boards (mainly deans/principals) to obtain their consent to collaborate, and then a trained multidisciplinary team (doctor, nurse, physical therapist, and physical education teachers) visited the pediatric clinic and schools to give presentations on the benefits of good health. After these educational sessions, we assessed all adolescents from the institutions for weight and height.

We included in this study all adolescents aged 12–16 years with a body mass index z-score (zBMI) ≥ + 2SD, puberty stage >3 [19]. The boys and girls had to present a minimum physical fitness to be included in this study. Therefore, we initially classified them according to the International Physical Activity Questionnaire (IPAQ), according to the activity performed during the last week, and confirmed this using the cardiopulmonary test (VO2peak). The participant was considered active when he/she performed five days or more of physical activity with a total of 150 min or more per week; irregularly active when not reaching the 150 min threshold; or sedentary when performing 10 min or less per week [19]. As all children included participated in physical activities at school, none was considered sedentary in this study, and all were fit. Those irregularly active substituted their extra activities outside school for the physical training proposed in this protocol.

We excluded individuals who lived in cities or states outside the coverage of the free transportation offered or with a time conflict between the sessions and the work of their companions or those undergoing drug treatment for weight control or who had heart, orthopedic, respiratory, or cardiac problems, kidney disease, diabetes, uncontrolled hypertension, genetic syndrome, or hormonal abnormalities.

### 2.4. Outcomes Evaluations

We evaluated these main outcomes in this study, before and after the intervention: anthropometric measures (weight and height), cardiorespiratory measures (as described in detail below), and the profile of lipid and glucose expenditure. One doctor, one nurse, one physiotherapist, and three physical education professionals were trained to conduct the assessments and reassessments in the institutions. The same examiners were designated to perform each specific procedure: always the same two for anamneses, one for anthropometric measures and body composition evaluations, and three for cardiopulmonary tests. The same professionals conducting the exercise sessions also performed evaluations. However, they were not researchers in this protocol, and they were blinded to the hypothesis of the study and to the insulin resistance status of each of the participants.

### 2.5. Anthropometry and Body Composition

We performed the anthropometric measures seven days before the intervention. For measuring body weight, participants assumed a standing position, wore light clothes and no footwear (mechanical scale, Filizola). We measured height using a stadiometer (meters). We calculated body mass index (BMI) and zBMI using the WHO AnthroPlus Software package (version 1.0.4, WHO, Geneva, Switzerland). We performed electrical bioimpedance (BIA, Biodynamics 310e, TBW Importer Ltd, Sao Paulo, Brazil), for the analysis of the percentage of body fat (%BF), fat mass (FM), fat-free mass (FFM), and basal metabolic rate (BMR) [19].

### 2.6. Lipid and Glucose Profile

Blood samples were drawn two days before the exercise intervention, after 12 h of fasting, for determination of total cholesterol (TC), high-density lipoprotein cholesterol (HDL-C) and triglycerides (TG), glucose, insulin and calculation of low density (LDL-C) and very high density (VLDL-C) cholesterol. Insulin resistance was determined by the Homeostatic Model Assessment for Insulin Resistance (HOMA-IR) formula: insulin (µUI/mL) × glucose (mmol/L)/22.5 [20].

For the final evaluation of the effects of exercising, one independent researcher divided the adolescents into two groups according to a HOMA-IR score cut-off of ≥3.16 [21]. Thus, we evaluated the participants in the groups insulin-resistant (IR) and insulin-sensitive (IS).

### 2.7. Cardiopulmonary Test

In the cardiopulmonary evaluation, the procedure for determining peak VO2, Vpeak, and VAT1 was: allowing 10 min of rest, followed by measurement of heart rate (HR; heart monitor model Sport Test; Polar Electro OY, Kempele, Finland), cardiac rhythm (digital electrocardiograph; MicroMed, Joinville, Brazil), blood pressure (BP; Classic II stethoscope, Littman, Portland, OR, USA; aneroid sphygmomanometer, Premium) and rating of perceived exertion (RPE). The cardiopulmonary test protocol included running on a treadmill (Model ATL, Inbrasport Ltd., Porto Alegre, Brazil) at a speed of 4 km/h with 1 km/h increments every minute until the participant showed exhaustion.

We evaluated ventilatory parameters on a gas analyzer (model VO2000; Inbrasport Ltd., Porto Alegre, Brazil). We recorded heart rate, cardiac rhythm, and RPE continuously. Upon exhaustion of the participant, we allowed two recovery periods of 2 min each, with 50% and 25% of peak velocity attained. During recovery periods, we recorded only the HR.

The criteria we used for determining VO2peak and exhaustion were: occurrence of VO2 plateau (characterized by 2 mL/kg/min increase) and being unable to maintain the running speed. Vpeak corresponded to the highest speed attained during the test. We determined VAT1 [22] using the V-slope method of Beaver et al. [23].

### 2.8. Indirect Calorimetry

To study the balance of energy substrates during exercise, we used indirect calorimetry measurement. Indirect calorimetry was performed after 4 h fasting, followed by ingestion of maltodextrin (0.5 g/kg, 10% solution) 30 min prior to the physical activity. The exercise protocol for this test consisted of 30 min of walking/running on a treadmill, split into 5 stages of 6 min each at intensities of 20, 30, 40, 50, and 60% VO2peak [24].

We determined lipid oxidation (LIPox) and CHOox rates based on mean values of VO2 and VCO2 (in L/min), corresponding to the last two minutes of each stage. We calculated the oxidation rate (in g/min) and energy derived from LIPox and CHOox (LIPkc and CHOkc, respectively; in kcal/min) using Frayn stoichiometric equations, assuming insignificant nitrogen excretion rate. The LIPkc and CHOkc, respectively, were calculated from their respective energy equivalents 9.75 and 3.87 kcal/g; for LIP and CHO, respectively [25].

### 2.9. Intervention: High-Intensity Exercise Training (HIT)

We trained the adolescents in six exercise sessions, three times a week, for two weeks [12,16,26,27]. We advised all participants to maintain their usual dietary patterns throughout the study period. A physiotherapist and four physical education teachers supervised the six HIT sessions.

The sessions took place in the physiology and metabolism laboratory of a university in the cardiorespiratory clinic of another university and a gym in São Paulo, both temperature and humidity-controlled environments. Participants performed the exercises always at day time (the lab worked from 8:00 h to 16:00 h) with a 48 h interval between sessions.

The protocol consisted of running on the treadmill. It comprised a two-minute warm-up at 25% Vpeak, followed by six series of 60 s runs at 100% Vpeak, intermixed with three-minute recoveries at 50% Vpeak, and two minutes of warm-down at 25%Vpeak [26].

### 2.10. Statistical Analysis

We carried out the statistical analysis using the Statistics for Windows software package (version 8.0, 2007; Statsoft, Inc., Tulsa, OK, USA) and considered a 5% level of significance. We observed data homogeneity using the Shapiro–Wilks and Levene tests, and therefore, we made comparisons of independent variables using the Mann–Whitney or unpaired Student’s *t*-test, whereas we used the Wilcoxon and Student’s *t*-test for dependent variables. We determined the magnitude of the HIT effect by Cohen’s *d* pooled method, with a confidence interval (CI), where: 0.20 < *d* > 0.49 = small effect; 0.5 < *d* > 0.79 = medium effect; 0.8 < *d* > 1.29 = large effect; and *d* > 1.30 = very large effect size.

## 3. Results

**Figure 1:**
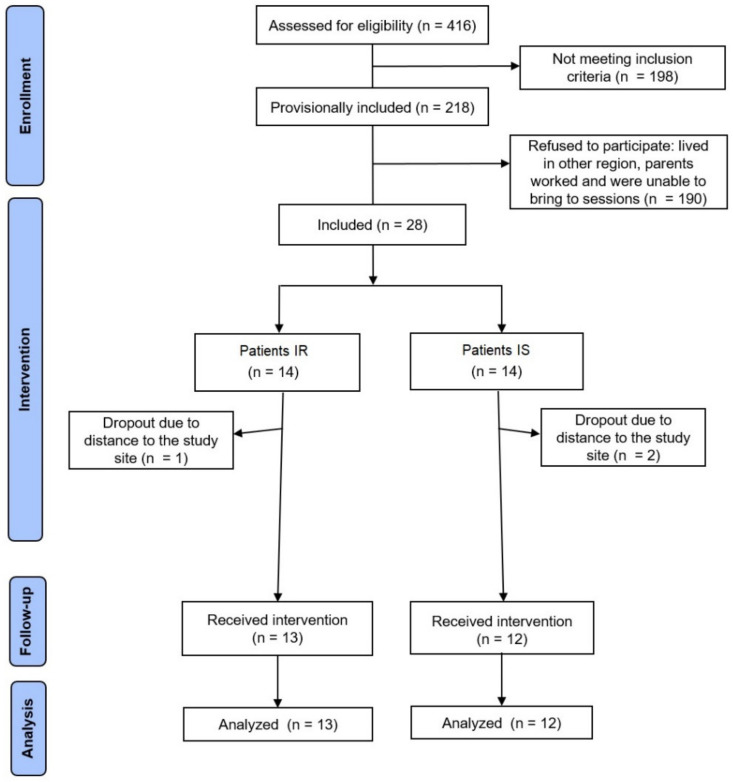
Flow chart depicting process of enrollment, allocation and follow-up of the 25 study participants. Groups insulin-resistant (IR) and insulin-sensitive (IS).

During the visits of the three schools, we evaluated the anthropometry of 416 adolescents, of whom 28 were considered obese and agreed to participate in this study. Among these, 14 had insulin resistance (IR group) and 14 did not (IS). The participants’ flow is shown in Figure 1.

Table 1 shows the baseline characteristics of the participants. During the period of evaluations, two individuals from the IS group and one individual from the IR group decided to drop out due to the distance between their homes and the evaluation sites.

Mean Vpeak for training was 8.85 ± 1.28 km/h (IR) and 9.08 ± 0.90 km/h (IS), with an intensity of 92.94% (IR) and 94.89% (IS) of HRpeak. No significant differences were observed for %HRpeak during training sessions or among groups.

Data on cardiorespiratory performance at pre and post-training for both groups are given in Table 2. The IR showed a 11.29% increase in Vpeak (*p* = 0.002) and 19.03% in V_VAT1_ (*p* = 0.005). The IS showed an 18.19% increase in VO2_VAT1_ (*p* = 0.021), 9.18% in HR_VAT1_ (*p* = 0.003), 12.88% in Vpeak (*p* = 0.001) and 26.28% in V_VAT1_ (*p* = 0.002).

No differences were observed among the groups after HIT for VO, V, and HR at peak intensity and VAT1. However, an effect was detected for VO2peak (*d* = 0.24; 95% CI from −3.06 to 5.46; *p* = 0.567), VO2VAT1 (*d* = 0.37; 95% CI from −3.71 to 9.51; *p*= 0.993), Vpeak (*d* = 0.29; 95% CI from −0.75 to 1.55; *p* = 0.473), VVAT1 (*d* = 0.47; 95% CI from −2.74 to0.998; *p* = 0.679); for HRpeak (*d* = 0.41; 95% CI from −5.95 to 17.61; *p* = 0.316); and clinical influence for HR_VAT1_ (*d* = 0.82; 95% CI from −0.12 to 12.68; *p* = 0.268).

**Figure 2:**
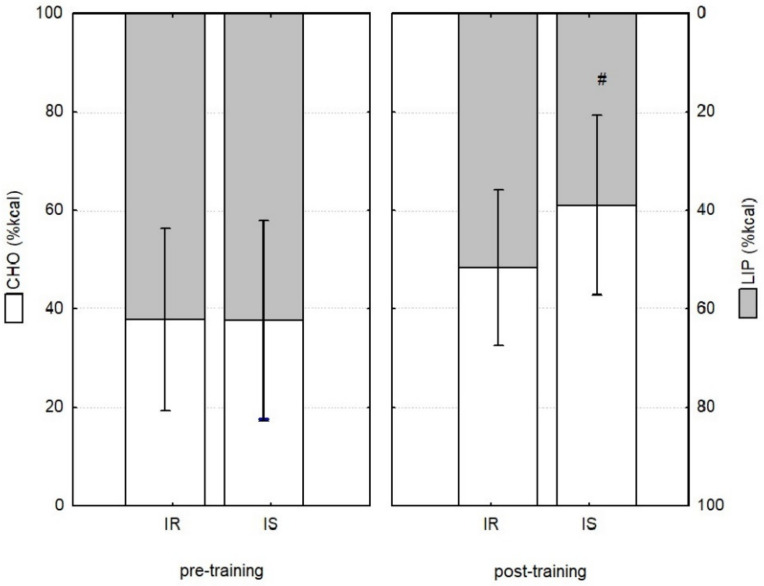
Energy derived from carbohydrates (CHO) and lipids (LIP) pre-and post-training. Mean values ± standard deviation; insulin-resistant (IR, n = 13) and insulin-sensitive (IS, n = 12). # Indicates *p* < 0.05 versus the period pre-training in the same group.

Prior to HIT, the CHOox and LIPox were proportional and similar among groups 62.15% (IR) and 62.30% (IS) of pre-training energy expenditure was derived from lipid metabolism (Figure 2).

The IR after HIT showed alterations in substrate oxidation (*p* > 0.05). In the IS, there was an increase in CHOox (56.13%, *p* = 0.039) and carbohydrate-derived energy (55.76%, *p* = 0.034), along with a reduction in LIPox (31.56%, *p* = 0.078) and lipid-derived energy (30.73%, *p* = 0.034).

No differences were observed among the groups after HIT for CHOox, LIPOox, and total oxidation (TTox). Analysis of the influence of HIT among the groups showed an effect for CHOox (*d* = 0.20; 95% CI from −7.20 to 10.50; *p* = 0.709), carbohydrate-derived energy (*d* = 0.20; 95% CI from 28.00 to 40.80; *p* = 0.000), total LIPox (*d* = 0.40; 95% CI from −1.60 to 5.40; *p* = 0.269) and lipid-derived energy (*d* = 0.50; 95% CI from −15.30 to 52.30; *p* = 0.000).

## 4. Discussion

In the present study, we observed a positive effect of the six HIT training sessions for the obese adolescents, both with and without insulin resistance. Participants showed improved fitness in both groups analyzed, increased carbohydrates oxidation, and contribution of this substrate to sustain energy needs in IS group.

Prior to the HIT, the volunteers had similar substrate oxidation patterns, contradicting the hypothesis that obese adolescents with insulin resistance would have greater limitations in carbohydrate metabolism. The limitation in CHOox detected in both groups of the present study may be associated with the low concentration of muscle glycogen, given that the content of this substrate relative to muscle mass is usually lower in children, adolescents, and the obese [28,29].

Another possible explanation of the results is that elevated intramuscular fatty acids (FA) can contribute to a reduction in blood glucose uptake and CHOox [30,31,32,33]. Braun et al. [34] noted that the FA re-esterification in obese subjects favors greater lipid availability during exercise, without implying increased oxidation. However, this greater availability would stimulate acetyl-CoA and citrate production, which leads to inhibition of hexokinase enzymes, phosphofructokinase and dehydrogenase pyruvate and consequently to a limitation in CHOox, as seen in the present study [31,32]. According to Hojlund [32], besides disturbances in oxidation, obesity is accompanied by alterations in the insulin signaling transduction pathway. These events limit the activation of glucose transporter isoform 4 (GLUTs 4) proteins, which explains the decline in glucose uptake in muscle and its oxidation.

The pre-HIT oxidation rates at the study baseline were similar to those reported by Barker et al. [18], although CHOox was limited in the volunteers of the present study. This finding suggests some inability to switch among the oxidative and glycolytic metabolisms, which were described as “metabolic inflexibility” [27,33,35], a phenomenon not specific to adults or associated with insulin resistance alone.

According to Tofiq et al. [36], insulin-resistant individuals are initially normoglycemic, such as the case in the present sample. However, long-term resistance and hypersecretion of the hormone can lead to failure of pancreatic beta cells, resulting in glucose intolerance, hyperglycemia, and subsequent diabetes mellitus type 2 [37]. This fact emphasizes the relevance of the present study.

The VO2peak values observed before training were lower than the recommended values for boys and girls [38,39], showing low fitness in both groups. This shows the importance of physical activity training programs for the pediatric population.

As expected, the HIT program promoted beneficial effects for other parameters such as Vpeak, VO2VAT1, and VVAT1. Indeed, the magnitude of improvement in some of these parameters was greater than those reported by Barker et al. [18], and similar to Corte de Araujo et al. [26], whose interventions comprised 30 and 36 training sessions, respectively, both designed for children and adolescents. Unfortunately, we cannot confirm the mechanisms involved in these adaptations. Jacobs et al. [40], however, in a study of untrained adults employing a similar HIT program, reported an increase in muscle oxidative capacity as a result of cytochrome-c-oxidase enzyme activity.

According to Racil et al. [41], changes in central factors, such as maximum cardiac output, total hemoglobin, and plasma volume, are affected by long-term HIT (>12 weeks), because longer interventions are needed to induce adaptations, explaining the absence of significant differences for VO2peak in the present study in the IR group after only six training sessions.

However, in the present study, we observed that the IS group, even in a short period of intervention, showed an improvement in VO2peak (increased by 10.99%), data that corroborate those presented by Gibala et al. [42], who after a short HIT program, observed an improvement in cardiorespiratory fitness associated, according to them, with peripheral adaptations, such as increased mitochondrial biogenesis and improved muscle buffering capacity. Marquezi et al. [13] verified that after six sessions of HIT on a treadmill ergometer there is a stimulus for mitochondrial biogenesis due to the increase in PGC-1α protein content, an adaptation similar to that observed after a single HIT session [43].

The results of the present study show that six sessions of HIT promoted an increase in CHOox in the IS group. This phenomenon can be explained by the greater sensitivity of alpha- and beta-adrenergic receptors, with increased glucose uptake, greater glycolytic enzyme activation, and modulation of substrate oxidation due to moderate-to-high strength or resistance exercise [44]. According to Jeppesen and Kiens [45], these adaptations are the result of greater recruitment of type II fibers and blood adrenaline concentrations, which stimulate glycogen degradation, glycolysis, and consequently, lactic acid production.

According to Kahlhöfer et al. [30], glucose metabolism produces pyruvate and forms acetyl-CoA through PDH. Acetyl-CoA condenses to oxaloacetate via the action of citrate synthase to form citrate, which is exported from the mitochondria by means of the action of ATP-citrate lyase. This forms acetyl-CoA, which is converted into malonyl-CoA, a potent inhibitor of the carnitine acetyltransferase complex which results in the inhibition of the oxidation of fatty acids in mitochondria.

HIT can induce alterations in substrate transport proteins, associated with the metabolism of glucose and FA. According to Burgomaster et al. [46], 18 sessions of HIT increased GLUT4 content and facilitated glucose uptake during recovery, with greater muscle glycogen levels observed after training. The authors added that several weeks of HIT can increase LIPox capacity in muscle, which is associated with greater hydroxyacyl-CoA dehydrogenase activity. However, such as the present study, the authors failed to observe any increase in LIPox post-training, suggesting that the adaptations to pathways associated with the metabolism of FA are processed more slowly. However, additional research is warranted to clarify the molecular mechanisms responsible for metabolic adaptation induced by these different acute exercise “impulses”. For example, HIT may differ from MICT with respect to changes induced in the cardiovascular and respiratory systems, metabolic control in other organs (liver or adipose tissue), and protection from various factors associated with chronic inactivity (insulin resistance, lipid dysregulation, or metabolic inflexibility) [27,33,35,46].

Energy expenditure for other physical activities, besides the training undertaken in the protocol, and the dietary intake of participants were not monitored, representing a limitation of the present study. Participants were instructed to maintain their normal everyday activities and diet. Therefore, the influence that uncontrolled activities and/or changes in the dietary pattern may have had on the parameters assessed is unclear.

One could argue that another limitation of the present study would be the inclusion of participants of both sexes, as influences of the menstrual cycle on the response to exercise cannot be ruled out. However, a study by Rossow et al. [47] showed that hemodynamic and cardiorespiratory responses to HIT do not differ among men and women. Moreover, Chu et al. [48] affirmed that, in overweight adolescents, there is minimal interaction among adiposity, pubertal status, gender, and substrate oxidation during exercise. However, the relationship between HIT and female characteristics is unclear in women of reproductive age. Thus, more research is needed to test the potential superiority of HIT vs. MICT on body composition and general oxidative metabolism, as well as to elucidate whether hormonal changes in the female ovarian cycle or the use of different types of contraceptives (estrogen and progesterone) could induce a state of “flexibility or inflexibility” metabolic effects in the female audience under different HIT training protocols [35].

Finally, the short period of training in this study might be considered an advantage of the intervention proposed. However, further studies involving cohorts exercising for longer periods should be conducted to explore the long-term effects of HIT on insulin-resistant obese adolescents. Another key variable in HIT research for teens is the volume of exercise HIT per session and the duration (number of sessions, minutes per week, and number of weeks) required for the prevention of complications in this age group.

## 5. Conclusions

We conclude that six HIT sessions improved cardiorespiratory performance in both groups and increased CHOox in insulin-sensitive obese adolescents, suggesting its utility for controlling physical fitness and glycemia in obese adolescents.

## Figures and Tables

**Table 1:** Baseline data of groups insulin-resistant (IR) and insulin-sensitive (IS).

Variables	IR (n = 13) Mean ± SD	IS (n = 12) Mean ± SD	*p* IR × IS
Age (years)	13.08 ± 1.66	12.75 ± 1.54	0.616
Weight (kg)	77.68 ± 10.66	69.11 ± 11.68	0.067
Height (m)	1.61 ± 0.07	1.58 ± 0.07	0.212
Body fat (%)	35.81 ± 4.28	30.41 ± 3.61	0.002
Fat mass (kg)	27.27 ± 6.61	20.41 ± 4.58	0.006
Fat-free mass (kg)	49.52 ± 6.55	46.46 ± 7.93	0.301
Basal metabolic rate (kcal)	1526.08 ± 192.50	1412.75 ± 240.59	0.205
BMI (kg/m^2^)	29.95 ± 4.33	27.37 ± 3.89	0.132
zBMI	2.59 ± 0.42	2.34 ± 0.58	0.218
Total cholesterol (mg/dL)	157.46 ± 43.43	156.54 ± 44.49	0.959
HDL-Cholesterol (mg/dL)	44.00 ± 9.73	47.00 ± 14.75	0.551
LDL-Cholesterol (mg/dL)	93.62 ± 33.53	93.00 ± 38.40	0.966
VLDL-Cholesterol (mg/dL)	19.54 ± 10.12	20.33 ± 6.10	0.816
Triglycerides (mg/dL)	97.69 ± 50.61	101.67 ± 30.48	0.816
Fasting glucose (mg/dL)	87.77 ± 5.82	87.00 ± 5.08	0.729
Fasting insulin (µUI/mL)	22.10 ± 6.45	9.83 ± 3.71	0.001
HOMA-IR	4.77 ± 1.33	2.11 ± 0.80	0.001

BMI: body mass index; zBMI: z-score of BMI; HDL-Cholesterol: high-density lipoprotein cholesterol; LDL-Cholesterol: low-density lipoprotein cholesterol; VLDL-Cholesterol very high-density lipoprotein cholesterol; HOMA-IR: insulin resistance index.

**Table 2:** Pre- and post-training mean values in the insulin-resistant (IR) group and insulin-sensitive (IS) group, for peak intensities and VAT1 and substrate oxidation during indirect calorimetry.

Variables	IR (n = 13) Mean ± SD				IS (n = 12) Mean ± SD			IR × IS
		Pre	Post	*p*	Pre	Post	*p*	*p*
VO2 (mL/kg/min)	Peak	26.82 ± 6.62	29.77 ± 6.08	0.064	30.62 ± 3.86	30.97 ± 3.88	0.583	0.567
	VAT1	14.77 ± 6.34	17.53 ± 4.21	0.125	14.84 ± 2.71	17.54 ± 3.24	0.021	0.993
HR (bpm)	Peak	190.69 ± 15.45	185.00 ± 16.99	0.063	187.33 ± 8.05	190.83 ± 10.39	0.186	0.316
	VAT1	143.62 ± 15.10	148.69 ± 16.54	0.397	129.83 ± 14.48	141.75 ± 13.82	0.003	0.269
V (km/h)	Peak	8.85 ± 1.28	9.85 ± 1.52	0.002	9.08 ± 0.90	10.25 ± 1.21	0.001	0.473
	VAT1	5.15 ± 0.90	6.31 ± 0.75	0.005	5.08 ± 0.29	6.42 ± 0.51	0.002	0.679
CHOox (g)		15.16 ± 17.54	18.21 ± 11.13	0.541	12.72 ± 10.84	19.86 ± 10.30	0.039	0.704
LIPox (g)		8.41 ± 4.56	7.29 ± 3.74	0.331	7.89 ± 3.89	5.40 ± 4.62	0.078	0.269
TTox (g)		23.57 ± 14.71	25.50 ± 8.56	0.652	20.50 ± 7.64	25.26 ± 7.30	0.042	0.939

SD: standard deviation; VO2: oxygen consumption; HR: heart rate; V: velocity; VAT1: exertion intensity at first ventilatory anaerobic threshold; CHOox: carbohydrate oxidation; LIPox: lipid oxidation; TTox: total oxidation.

## Data Availability

The data presented in this study are available on request from the corresponding author. The data are not publicly available due to privacy.
